# Joint Target Tracking, Recognition and Segmentation for Infrared Imagery Using a Shape Manifold-Based Level Set

**DOI:** 10.3390/s140610124

**Published:** 2014-06-10

**Authors:** Jiulu Gong, Guoliang Fan, Liangjiang Yu, Joseph P. Havlicek, Derong Chen, Ningjun Fan

**Affiliations:** 1 School of Mechatronical Engineering, Beijing Institute of Technology, No. 5, Zhongguancun South Street, Haidian District, Beijing 100081, China; E-Mails: gongjiulu@gmail.com (J.G.); cdr@bit.edu.cn (D.C.); njfan@bit.edu.cn (N.F.); 2 School of Electrical and Computer Engineering, Oklahoma State University, 202 Engineering South, Stillwater, OK 74078, USA; E-Mail: liangjiang.yu@okstate.edu; 3 School of Electrical and Computer Engineering, University of Oklahoma, 110 West Boyd, DEH 150 Norman, OK 73019, USA; E-Mail: joebob@ou.edu

**Keywords:** automatic target recognition, joint tracking recognition and segmentation, shape manifolds, level set, manifold learning

## Abstract

We propose a new integrated target tracking, recognition and segmentation algorithm, called ATR-Seg, for infrared imagery. ATR-Seg is formulated in a probabilistic shape-aware level set framework that incorporates a joint view-identity manifold (JVIM) for target shape modeling. As a shape generative model, JVIM features a unified manifold structure in the latent space that is embedded with one view-independent identity manifold and infinite identity-dependent view manifolds. In the ATR-Seg algorithm, the ATR problem formulated as a sequential level-set optimization process over the latent space of JVIM, so that tracking and recognition can be jointly optimized via implicit shape matching where target segmentation is achieved as a by-product without any pre-processing or feature extraction. Experimental results on the recently released SENSIAC ATR database demonstrate the advantages and effectiveness of ATR-Seg over two recent ATR algorithms that involve explicit shape matching.

## 1. Introduction

As a challenging problem in pattern recognition and machine learning for decades, automatic target tracking and recognition (ATR) has been an important topic for many military and civilian applications. Infrared (IR) ATR is a more challenging problem due to two main reasons. First, an IR target's appearance may change dramatically under different working conditions and ambient environment. Second, the IR imagery usually has poor quality compared with the visible one. There are two important and related research issues in ATR research, appearance representation and motion modeling [1]. The former one focuses on capturing distinct and salient features (e.g., edge, shape, texture) of a target, and the latter one tries to predict the target's state (e.g., position, pose, velocity) during sequential estimation. They could play a complementary role in an ATR process [2].

Shape is a simple yet robust, feature for target representation in many ATR applications. There are three commonly used ways of shape representation: a 3D mesh model [3], 2D shape templates [4,5] and a manifold-based shape generative model learned from 2D snapshots [6–8]. When a 3D model was used, a 3D-to-2D projection is needed to get the 2D shapes according to the camera model and the target's position. Using a 3D model for shape modeling usually needs more memory and expensive computational resources. In [5], a 2D shape template was used to represent the target's appearance, and an online learning was used to update this shape model under different views. Manifold learning methods have proven to be powerful for shape modeling by providing a variety of meaningful shape prior to assist or constrain the shape matching process. In [8], a couplet of view and identity manifolds (CVIM) was proposed for multi-view and multi-target shape modeling, where target pre-segmentation was implemented via background subtraction and the ATR inference involves explicit shape matching between segmented targets and shapes hypothesis generated by CVIM.

In this work, we propose a new particle filter-based ATR-Seg (segmentation) algorithm that integrates JVIM (joint view-identity manifold) with a shape-aware level set energy function which leads to a joint tracking, recognition and segmentation framework. JVIM encapsulates two shape variables, identity and view, in a unified latent space, which is embedded with one view-independent identity manifold and infinite identity-dependent view manifolds. Unlike CVIM obtained via nonlinear tensor decomposition, JVIM is learned via a modified Gaussian process latent variable model [9] which leads to a probabilistic shape model. Also, a stochastic gradient descent method [10] is developed to speed up JVIM learning, and a local approximate method is used for fast shape interpolation and efficient shape inference. Furthermore, we integrate JVIM with a level set energy function that is able to evaluate how likely a shape synthesized by JVIM can segment out a valid target from an image. This energy function is adopted as the likelihood function in the particle filter where a general motion model is used for handling highly maneuverable targets. The performance of ATR-Seg was evaluated using the SENSIAC (Military Sensing Information Analysis Center) IR dataset [11], which demonstrated the advantage of the proposed method over several methods that involve target pre-segmentation and explicit shape matching.

The remainder of this paper is organized as follow. In Section 2, we review some related works on shape manifold learning and shape matching. In Section 3, we use a graphical model to develop a probabilistic framework of our ATR-Seg algorithm. In Section 4, we introduce JVIM for general shape modeling. In Section 5, we present a shape-aware level set energy function for implicit shape matching.

In Section 6, we present a particle filter-based sequential inference method for ATR-Seg. In Section 7, we evaluate the proposed ATR-Seg algorithm in two aspects, *i.e.*, JVIM-based shape modeling and implicit shape matching which are involved in the likelihood function of the particle filter. We conclude our paper in Section 8.

## 2. Related Works

ATR itself is a broad field involving diverse topics. Due to the fact that shape modeling is the key issue in our ATR research, our review below will be focused on two shape-related topics, manifold-based shape modeling and shape matching.

### 2.1. Manifold-Based Shape Modeling

A manifold-based shape model can be learned from a set of exemplar shapes and is able to interpolate new shapes from the low-dimensional latent space. Roughly speaking, there are three manifold learning approaches for shape modeling, geometrically-inspired methods, latent variable models, and hybrid models. The first approach seeks to preserve the geometric relationships among the high-dimensional data in the low-dimensional space, e.g., IsoMap [12], Local Linear Embedding (LLE) [13], Diffusion Maps [14] and Laplacian Eigenmaps [15]. These methods focus on how to explore the geometric structure among the high-dimensional data and how to maintain this structure in the low dimensional embedding space. However, the mapping relationship from the latent space and the data space is not available and has to be learned separately. The second approach represents the shape data by a few latent variables along with a mapping from the latent space to the data space, such as PCA [16], PPCA [17], KPCA [18], Gaussian Process Latent Variable Models (GPLVM) [19] and tensor decomposition [20], *et al*. GPLVM is a probabilistic manifold learning method which employs the Gaussian process as the nonlinear mapping function. Above approaches are data driven shape modeling methods without involving prior knowledge in the latent space, and as a result, the shape-based inference process may be less intuitive due to the lack of a physically meaningful manifold structure.

To support a more meaningful and manageable manifold structure while preserving the mapping function, there is a trend to combine the first two approaches along with some topology prior for manifold learning [21]. In [9], the local linear GPLVM (LL-GPLVM) was proposed for complex motion modeling, which incorporates a LLE-based topology prior in the latent space. Specifically, a circular-shaped manifold prior is used to jointly model both “walking” and “running” motion data in a unified cylinder-shaped manifold. In [8], CVIM was proposed for shape modeling via nonlinear tensor decomposition where two independent manifolds, an identity manifold and a view manifold, were involved. Specifically, the view manifold was assumed to be a hemisphere that represents all possible viewing angles for a ground target, and the identity manifold was learned from the tensor coefficient space that was used to interpolate “intermediate” or “unknown” target types from known ones. A key issue about the identity manifold is the determination of manifold topology, *i.e.*, the ordering relationship across all different target types. Sharing a similar spirit of IsoMap, the shortest-closed-path is used to find the optimal manifold topology that allows targets with similar shapes to stay closer and those with dissimilar shapes far away. This arrangement ensures the best local smoothness and global continuity that are important for valid shape interpolation along the identity manifold.

### 2.2. Shape Matching

In shape-based tracking algorithms, there are two ways to measure shape similarity: explicit shape matching and implicit shape matching. The former one involves a direct spatial comparison between two shapes, an observed one and a hypothesized one, by using a certain distance metric. In such a case, pre-processing or feature extraction, e.g., background subtraction in [8], is needed prior to tracking and recognition, which is relatively manageable for a stationary sensor platform and may need additional computational load in a general case. Moreover, the overall ATR performance could be sensitive to the pre-processing results. The latter one represents a shape implicitly by a level set embedding function which can be used to evaluate the segmentation quality of a given shape in an image. For example, a shape-constrained energy function was used in [6,7] to evaluate how likely the given shape can segment out a valid object, where a gradient descent method was used to optimize this energy function to achieve tracking and segmentation jointly. Therefore, implicit shape matching does not involve any pre-processing or feature extraction beforehand, however, due to the lack of dynamic modeling in level set optimization, it is still hard to track highly maneuverable targets by the traditional data-driven gradient descent optimization method. As pointed in [22], motion/dynamic modeling is an important step for most ATR applications. This motivates our research to augment a motion model in implicit shape matching for maneuverable target tracking.

## 3. ATR-Seg Problem Formulation

**Figure 1.**
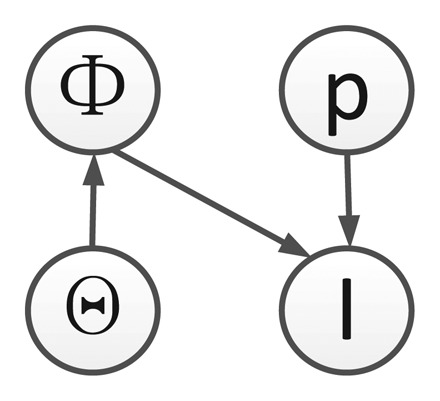
Graphical modeling for the proposed ATR-Seg algorithm, where **I***_t_* represents an image frame, **p** 3D target position, **Φ** target segmentation, and **Θ** the set of shape variables.

We list all symbols used in this paper in Table 1. Given the observed video sequence **I***_t_*, with *t* = 1,…, *T* , where T is the total number of image frames, the objective of ATR-Seg is to (1) find the 3D position of a target in the camera coordinate **p** (tracking) or 2D image coordinate, (2) to identify the target type *α* (recognition), along with the view angle *φ* (pose estimation), and (3) to segment the target-of-interest that best explains the observation data **Φ** (segmentation). The 2D shape of a target can be determined by the target type *α*, and view angle *φ*, so we define **Θ** = [*α*, *φ*] to represent two shape related variables. The conditional dependency among all variables is shown in Figure 1.

According to Figure 1, we define the objective function of ATR-Seg from the joint distribution *p*(**p***_t_*, **Θ***_t_*, **Φ**, **I***_t_*) which can be written (*t* is omitted for simplicity) as:

(1) p(p,Θ,Φ,I)=p(I|p,Φ)p(Φ|Θ)p(Θ)p(p)

By using the Bayesian theorem, we can get the posterior as:

(2) p(p,Θ,Φ,I)=p(I|p,Φ)p(Φ|Θ)p(Θ)p(p)

which encapsulates three major components in the proposed ATR-Seg algorithm, as shown below:

- Shape manifold learning provides a mapping from **Θ** to **Φ**, *i.e.*, *p*(**Φ**|**Θ**). In Section 4, JVIM is proposed for multi-view and multi-target shape modeling, which features a novel manifold structure with one view-independent identity manifold and infinite identity-dependent view manifolds to impose a conditional dependency between the two shape-related factors, view and identity, in a unified latent space.
- Shape-aware level set *p*(**I**|**p**, **Φ**) measures how likely **Φ** can segment a valid target at position **p** in image *I*. In Section 5, a shape-aware level set energy function is proposed for implicit shape matching, which evaluates the segmentation quality.
- Pose/position priors **Θ** and **p**, *i.e.*, *p*(**Θ**) and *p*(**p**), *i.e.*, are important to track highly manoeuverable targets in a sequential manner. In Section 6, sequential shape inference method is presented that involve dynamic priors for **Θ** and **p** using a 3D motion model.

**Figure 2.**
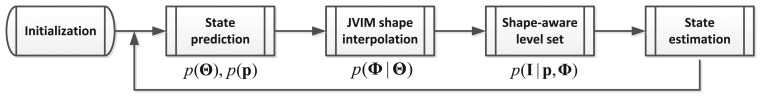
Flowchart for ATR-Seg.

The flowchart for ATR-Seg is shown in Figure 2 where four steps are involved sequentially and recursively. First, state prediction will draw a set of samples to predict all state variables (position/angle/identity). Second, a series of shape hypotheses are created via JVIM in some hypothesized locations according to predicted state information. Third, a level-set energy function is used as the likelihood function to weight each hypothesized shape/location that quantifies how well that shape can segment a valid target in that particular location. Fourth, state estimation at the current frame is obtained by the conditional mean of all weighted samples and will be used for state prediction in the next frame.

## 4. Joint View-Identity Manifold (JVIM)

**Figure 3.**
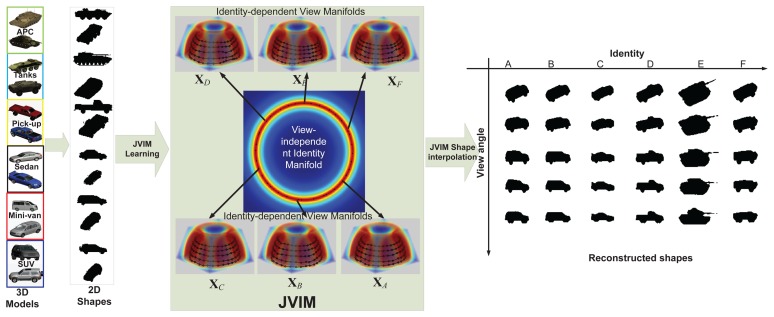
JVIM learning and shape interpolation, where one view-independent identity manifold and six identity-dependent view manifolds are color-coded according to the uncertainty of GP mapping. (Adapted from [24], with permission from Elsevier.)

JVIM is learned from a set of 2D shape exemplars **Y** generated from a set of 3D CAD models. The latent space **X** can be represented by two variables, identity *α* and view *φ* (including aspect angle *θ* and elevation angle *ϕ*), which are defined along their respective manifolds. Considering the fact that all targets have different 3D structures, leading to different view manifolds, and they keep the same identity under different views, we impose a conditional dependency between *α* and *φ* in JVIM that encapsulates one view-independent identity manifold and infinite identity-dependent view manifolds. Specifically, the identity manifold represents the view-independent shape variability across different target types, and an identity-specific view manifold captures the shape variability of a target under different views. Motivated by [8,23], the identity manifold is simplified to have a circular-shaped topology prior, which facilitates manifold learning and shape inference. Intuitively, a hemispherical-shaped topology prior is assumed for identity-specific view manifold, which represents all possible aspect and elevation angles for ground vehicle. All topology priors are encoded by LLE and incorporated into the GPLVM-based learning framework, as shown in Figure 3.

The objective of JVIM learning is to find **X** and *β* by maximizing *p*(**Y**|**X**, *β*, **w**), where *β* is the mapping parameter and w represents the LLE-based topology prior in the latent space. The Gaussian process (GP) is used as the nonlinear mapping function from the latent space to the shape space (**X** → **Y**), and the objective function of JVIM learning is written as:

(3) p(Y|X,β,w)=p(Y|X,β)p(X|w)

where:

(4) $$p(\text{Y}|\text{X},\beta )\frac{1}{\sqrt{{\left(2\pi \right)}^{Nd}|{\text{K}}_{Y}{|}^{d}}}\exp(-\frac{1}{2}tr({\text{K}}_{Y}^{-1}{\text{YY}}^{T}))$$

where *d* is the dimension of the shape space and *β* denotes the kernel hyper-parameters used in the covariance matrix, **K***_Y_*. It is worth noting that Equation (4) is similar to the objective function of GPLVM [19], and:

(5) $$p(\text{X}|\text{w})=\frac{1}{Z}\exp\left(-\frac{1}{\sigma^{2}}\sum_{i=1}^{N}∥{\text{X}}_{i}-\sum_{j=1}^{N}{\text{w}}_{ij}{\text{X}}_{j}{∥}^{2}\right)$$

where **w** is the set of LLE weights to reconstruct each latent point from its local neighboring points by minimizing $f(\text{w})=\sum_{i=1}^{N}∥{\text{X}}_{i}-\sum_{j=1}^{N}{\text{w}}_{ij}{\text{X}}_{j}{∥}^{2}$, *Z* is a normalization constant, *σ*^2^ represents a global scaling of the prior and *N* the number of training samples. Furthermore, the negative log operation is used to simplify the objective function as:

(6) $$L_{\text{JVIM}}=-\log p(\text{Y}|\text{X},\beta )p(\text{X}|\text{w})=L_{D}+L_{T}+C$$

where *C* is a constant.

JVIM learning involves a gradient descent method to minimize the objective function defined in Equation (3) with respect to **X** and *β*. With an *O*(*N*^3^) operation required at each iteration, it is computationally prohibitive for a large training data set. The stochastic gradient descent proposed in [10] is adapted to be a local updating according to the unique structure of JVIM to approximate the gradients locally. At each iteration, the reference point, **x***_r_*, is chosen randomly, and the derivatives w.r.t **X***_R_* and *β* are calculated as:

(7) $$\frac{\partial L_{D}}{\partial {\text{X}}_{R}}\approx -({\text{K}}_{R}^{-1}{\text{Y}}_{R}{\text{Y}}_{R}^{T}{\text{K}}_{R}^{-1}-d{\text{K}}_{R}^{-1})\cdot \frac{\partial {\text{K}}_{R}}{\partial {\text{X}}_{R}}$$

(8) $$\frac{\partial L_{\text{JVIM}}}{\partial \beta }\approx -({\text{K}}_{R}^{-1}{\text{Y}}_{R}{\text{Y}}_{R}^{T}{\text{K}}_{R}^{-1}-d{\text{K}}_{R}^{-1})\cdot \frac{\partial {\text{K}}_{R}}{\partial \beta }$$

where **X***_R_* is the neighborhood for a reference point, **x***_r_*, of size *M*_1_, **Y***_R_* is the corresponding shape data and **K***_R_* (*M*_1_ × *M*_1_) is the kernel matrix of **X***_R_*. The neighborhood for each training data can be pre-assigned according to the topology structure, and the gradients are estimated stochastically, locally and efficiently.

As a generative model, given an arbitrary latent point in **X**, JVIM can generate the corresponding shape via Gaussian Process (GP) mapping. For real-time applications, shape interpolation must be carried out efficiently, which is difficult for a large training data set with high dimensionality. Inspired by [25], a GP can be approximated by a set of local GPs, in JVIM-based shape interpolation, the kernel matrix is computed locally from a set of training data that are close to the given point. Given **x′**, we first find its closest training point, which has a pre-assigned neighborhood, **X′**, of size *M*_2_; then, **X′** and the corresponding shape data **Y′** are used to approximate the mean and variance of GP mapping as:

(9) $${\hat{\mu }}_{x^{\prime }}={\text{k}}_{x^{\prime }X^{\prime }}^{T}{\text{K}}_{Y^{\prime }}^{-1}{\text{Y}}^{\prime }$$

(10) $${\hat{\sigma }}_{x^{\prime }}^{2}=k({\text{x}}^{\prime },{\text{x}}^{\prime })-{\text{k}}_{x^{\prime }X^{\prime }}^{T}{\text{K}}_{Y^{\prime }}^{-1}{\text{k}}_{x^{\prime }X^{\prime }}$$

where k*_x′X′_* is a vector made of *k*(**x′**, **x***_i_*) (**x***_i_* ∈ **X′**) and **K***_Y′_* (*M*_2_ × *M*_2_) is the local covariance matrix computed from **X′**. More detail about JVIM learning and inference can be found in our previous work [26], where explicit shape matching is involved. In the following section, we will introduce implicit shape matching by incorporating a shape-aware level set for target tracking and recognition, where target segmentation becomes a by-product.

## 5. Shape-Aware Level Set

JVIM is used to provide a useful shape prior that can be further combined with the level set to define an energy function for implicit shape matching. This is called the shape-aware level set, which does not involve feature extraction or target pre-segmentation. The shape-aware level set in this work is distinct from that in [6,7,27] primarily in two aspects. Firstly, the shape generated model in [6,7], which was less structured with little semantic meaning and, was limited to object recognition/segmentation under the same view or human pose estimation for the same person along the same walking path. JVIM is a multi-view and multi-target shape model that has a well-defined semantic structure, which supports robust ATR for different targets under arbitrary view angles. Secondly, a gradient decent method was used for level set optimization in [6,7,27], which does not involve a motion model and makes it hard to track highly maneuverable targets. In this work, a 3D motion model is used to combine the position/pose priors into a sequential optimization model to improve the robustness and accuracy of ATR-Seg.

**Figure 4.**
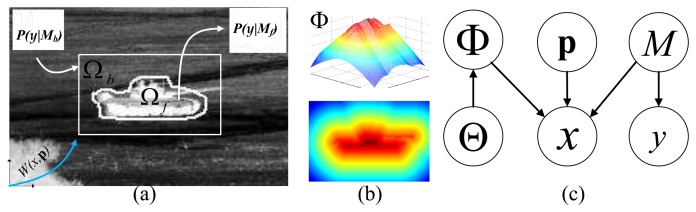
Shape-aware level set model for implicit shape matching. (**a**) Illustration of a target in an infrared image: foreground Ω*_f_* and background Ω*_b_*, foreground/background intensity models *M*, and the 3D-2D camera projection W(x,p). (**b**) The shape embedding function **Φ**. (**c**) The graphical model for shape-aware level set, where **p** is the target 3D location of a ground-vehicle, and **Θ** is the shape parameter in JVIM.

As shown in Figure 4a, we represent an image by **I** = {*x_i_*, *y_i_*}, where 1 ≤ *i* ≤ *n*, *n* is the number of pixels in I and *x* and *y* are the pixel 2D location and pixel intensity value, respectively. We introduce a parameter, *M*, to represent the foreground/background models *M* = {*M_f_*, *M_b_*}; then, the original graphical model of ATR-Seg in Figure 1 will become the one in Figure 4c. which defines a joint distribution of all parameters for each pixel (*x_i_*, *y_i_*) as

(11) $$p(x_{i},y_{i},\text{p},\text{Θ},\text{Φ},M)=p(x_{i}|\text{p},\text{Φ},M)p(y_{i}|M)p(\text{Φ}|\text{Θ})p(M)p(\text{Θ})p(\text{p})$$

where **Φ** is a shape represented by the level set embedding function shown in Figure 4b and *p*(**Φ**|**Θ**) corresponds to JVIM-based shape interpolation via GP mapping. A histogram is used for foreground/background appearance model *p*(*y_i_*|*M*), and the number of bins is dependent on the size of the target and gray scale. In order to get the posterior, *p*(**p**, **Θ**, **Φ**, *M*|*x_i_*, *y_i_*), which will be used to develop the objective function for ATR-Seg, we take the same strategy as in [27]. First, divide Equation (11) by $p(y_{i})=\sum_{j\in \{f,b\}}p(y_{i}|M_{j})p(M_{j})$:

(12) $$p(x_{i},\text{p},\text{Θ},\text{Φ},M|y_{i})=p(x_{i}|\text{p},\text{Φ},M)p(M|y_{i})p(\text{Φ}|\text{Θ})p(\text{Θ})p(\text{p})$$

where *p*(*M*|*y_i_*) is given by:

(13) $$\begin{array}{cc} p(M_{j}|y_{i})=\frac{p(y_{i}|M_{j})p(M_{j})}{\sum_{k\in \{f,b\}}p(y_{i}|M_{k})p(M_{k})}, & j\in \{f,b\} \end{array}$$

Upon dividing Equation (12) by *p*(*x_i_*) = 1/*n* and marginalizing over the models, *M*, we obtain:

(14) $$p(\text{p},\text{Θ},\text{Φ}|x_{i},y_{i})=n\sum_{j\in \{f,b\}}p(x_{i}|\text{p},\text{Φ},M_{j})p(M_{j}|y_{i})p(\text{Φ}|\text{Θ})p(\text{Θ})p(\text{p})$$

Assuming all pixels are independent, the posterior for all pixels in a frame is then given by:

(15) $$\begin{array}{l} p(x_{i}|\text{p},\text{Φ},M_{f})=H_{\epsilon }[\text{Φ}(x_{i})]/\eta_{f}\\ p(x_{i}|\text{p},\text{Φ},M_{b})=\{1-H_{\epsilon }[\text{Φ}(x_{i})]\}/\eta_{b} \end{array}$$

where *H_ϵ_* [·] is the smoothed Heaviside step function, $\eta_{f}=\sum_{i=1}^{n}H_{\epsilon }[\text{Φ}(x_{i})]$, $\eta_{b}=\sum_{i=1}^{n}\{1-H_{\epsilon }[\text{Φ}(x_{i})]\}$ and *p*(*M_j_*) = *η_j_*/*n* for *j* ∈ {*f*, *b*}.

**Figure 5.**
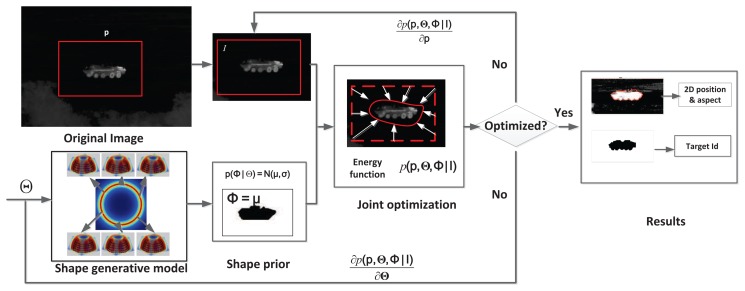
Optimization of ATR-Seg by a gradient descent method.

Then, from Equations (2), (14) and (15), we have:

(16) $$p(\text{I}|\text{p},\text{Φ})\propto \prod_{i=1}^{n}\sum_{j\in \{f,b\}}p(x_{i}|\text{p},\text{Φ},M_{j})p(M_{j}|y_{i})$$

which evaluates how likely shape **Φ** can segment a valid target from **I** at position **p**. The objective function in Equation (2) can be optimized through a gradient descent method similar to the one in [7], which is illustrated in Figure 5. As shown in Figure 5, JVIM is firstly used to generate a shape hypothesis, **Φ**^0^, given initial identity and view angle **Θ**^0^; then, **Φ**^0^ is used to initialize the objective function, *p*(**p**, **Θ**, **I**), for initial position, **p**^0^. We take the derivative of *p*(**p**, **Θ**, **Φ**|**I**) with respect to **Θ** and **p** to get $\frac{\partial p(\text{p},\text{Θ},\text{Φ}|\text{I})}{\partial \text{Θ}}$ and $\frac{\partial p(\text{p},\text{Θ},\text{Φ}|\text{I})}{\partial \text{p}}$, which will be used to update **Θ** and **p** until the objective function converges. When *p*(**p**, **Θ**, **Φ**|**I**) is maximized, we output the updated target's 2D position, p*, target identity and view angle Θ*, as well as the updated shape Φ* that can best segment the image.

**Figure 6.**
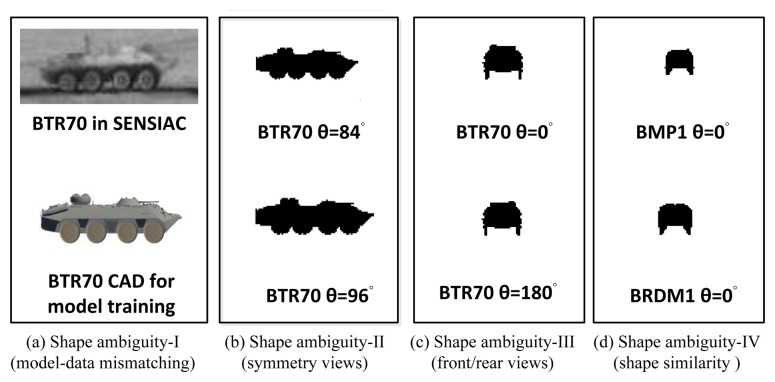
Possible reasons for the failure of the gradient descent method.

This method works well on a single image when a good initialization is given in the latent space of JVIM. However, it may fail quickly when dealing with an image sequence with a highly maneuverable target, due to four possible cases of shape ambiguity, as shown in Figure 6, which makes data-driven optimization not reliable in practice. (1) The first is due to the possible shape mismatch between the CAD models and real targets, even for the same target type (Figure 6a). (2) The second is due to the symmetry property of a target's shape (Figure 6b), which means a target may present a similar shape at different (e.g., supplement) aspect angles, especially when the elevation angle is zero (Figure 6b). (3) The third is due to the ambiguity of the front/rear views when a target looks very similar (Figure 6c). (4) The fourth is similar to the previous one in which many targets look alike at the front/rear views (Figure 6d). These factors make the gradient-based approach not effective at dealing with a maneuvering target. A possible remedy is to introduce a dynamic motion model to support robust sequential shape inference based on JVIM, as to be discussed below.

## 6. Sequential Shape Inference

Essentially, the objective of ATR-Seg is to perform sequential shape inference from an image sequence by maximizing the posterior of *p*(**p***_t_*, **Θ***_t_*, **Φ***_t_*|**I***_t_*). According to Figure 1 in Section 3, **Φ** is only dependent on **Θ**, so the objective function can be rewritten as:

(17) $$p({\text{p}}_{t},{\text{Θ}}_{t},{\text{Φ}}_{t}|{\text{I}}_{t})=p({\text{p}}_{t},{\text{Θ}}_{t}|{\text{I}}_{t})p({\text{Φ}}_{t}|{\text{Θ}}_{t})$$

where *p*(**Φ***_t_*|**Θ***_t_*) is JVIM-based shape interpolation via GP mapping. Since *p*(**Φ***_t_*|**Θ***_t_*) is not related to the observation, so the main computational load is the maximization of *p*(**p***_t_*, **Θ***_t_*|**I***_t_*). For sequential ATR-Seg, the optimization of *p*(**p***_t_*, **Θ***_t_*|**I***_t_*) has two stages: prediction and update. In the first stage (prediction), we use a motion model to predict *p*(**p***_t_*, **Θ***_t_*|**I***_t_*_−1_) from the previous result *p*(**p***_t_*_−1_, **Θ***_t_*_−1_|**I***_t_*_−1_) as:

(18) $$p({\text{p}}_{t},{\text{Θ}}_{t}|{\text{I}}_{t-1})=\int \int p({\text{p}}_{t-1},{\text{Θ}}_{t-1}|{\text{I}}_{t-1})p({\text{p}}_{t}|{\text{p}}_{t-1})p({\text{Θ}}_{t}|{\text{Θ}}_{t-1})d{\text{Θ}}_{t-1}d{\text{p}}_{t-1}$$

where *p*(**p***_t_*|**p***_t_*_−1_) and *p*(**Θ***_t_*|**Θ***_t_*_−1_) are used to predict the position and identity/view of a moving target. They are related a motion model that characterizes the target's dynamics and kinematics. In the second stage (update stage), we use the Bayes' rule to compute the posterior as:

(19) $$p({\text{p}}_{t},{\text{Θ}}_{t},{\text{Φ}}_{t}|{\text{I}}_{t})=\frac{p({\text{p}}_{t},{\text{Θ}}_{t},{\text{Φ}}_{t}|{\text{I}}_{t-1})p({\text{I}}_{t}|{\text{p}}_{t},{\text{Φ}}_{t})}{p({\text{I}}_{t}|{\text{I}}_{t-1})}$$

where *p*(**p***_t_*, **Θ***_t_*, **Φ***_t_*|**I***_t_*_−1_) = *p*(**p***_t_*,**Θ***_t_*|**I***_t_*_−1_)*p*(**Φ***_t_*|**Θ***_t_*) and we have *p*(**I***_t_*|**p***_t_*, **Φ***_t_*, **Θ***_t_*) = *p*(**I***_t_*|**p***_t_*, **Φ***_t_*). Hence, the objective function of the sequential ATR-Seg algorithm can be further rewritten as:

(20) $$p({\text{p}}_{t},{\text{Θ}}_{t},{\text{Φ}}_{t}|{\text{I}}_{t})\propto p({\text{I}}_{t}|{\text{p}}_{t},{\text{Φ}}_{t})p({\text{Φ}}_{t}|{\text{Θ}}_{t})\int \int p({\text{Θ}}_{t}|{\text{Θ}}_{t-1})p({\text{p}}_{t-1},{\text{Θ}}_{t-1},{\text{Φ}}_{t-1}|{\text{I}}_{t-1})p({\text{p}}_{t}|{\text{p}}_{t-1})d{\text{Θ}}_{t-1}d{\text{p}}_{t-1}$$

**Figure 7.**
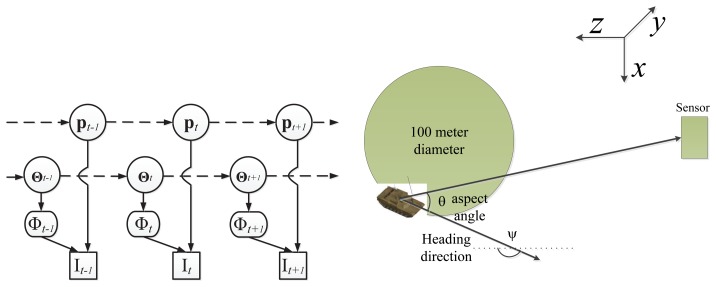
The graphical model representation of ATR-Seg and the 3D camera coordinate. (Reprint from [28] with permission from IEEE).

Due to the nonlinear nature of Equation (20), we resort to a particle filter-based inference framework [29] for sequential optimization, as represented by the graphic model in Figure 7 (left). Thanks to the compact and continuous nature of JVIM, we can draw samples from its latent space for efficient shape interpolation. In the inference process, the state vector is defined as ${\text{Z}}_{t}={\left[{\text{p}}_{t}^{T},\upsilon_{t},\psi_{t},\alpha_{t}\right]}^{T}$ where ${\text{p}}_{t}={\left[p_{x}^{t},p_{y}^{t},p_{z}^{t}\right]}^{T}$ represents the target's 3D position, with the *x* − *y* − *z* axes denoting the horizon (*x*), elevation (*y*) and range (*z*) directions, respectively (as shown in Figure 7 (right)); *υ_t_* is the velocity along the heading direction, *ψ_t_*. A 3D-2D camera projection, *W* (**p**), is needed to project a 3D position to a 2D position in an image that is assumed to be unchanging for a stationary sensor platform. It is worth noting that we can compute *θ_t_* (the aspect angle) from *ψ_t_* (the heading direction) or *vice versa*. As a matter of fact, the two angles are similar for distant targets when the angle between the lineof sight and the optical axis along the range direction (*z*) is very small. Because the target is a ground vehicle and to keep it general, a white noise acceleration model is used to represent the dynamics of **Z***_t_*, where a simple random walk is applied on the heading direction, *ψ_t_*, to represent arbitrary maneuvering. Moreover, we define the dynamics of *α_t_* (target identity) to be a simple random walk along the identity manifold by which the estimated identity value normally quickly converges to the correct one.

(21) $$\{\begin{array}{ll} \psi_{t} & =\psi_{t-1}+\zeta_{t}^{\psi },\\ \upsilon_{t} & =\upsilon_{t-1}+\zeta_{t}^{\upsilon },\\ p_{x}^{t} & =p_{x}^{t-1}+\upsilon_{t-1}\sin(\psi_{t-1})\Delta t+\zeta_{t}^{x},\\ p_{y}^{t} & =p_{y}^{t-1}+\zeta_{t}^{y},\\ p_{z}^{t} & =p_{z}^{t-1}+\upsilon_{t-1}\cos(\psi_{t-1})\Delta t+\zeta_{t}^{z},\\ \alpha^{t} & =\alpha^{t-1}+\zeta_{t}^{\alpha }, \end{array}$$

where Δ*t* is the time interval between two adjacent frames. The process noises associated with the target kinematics, $\zeta_{t}^{\psi }$, $\zeta_{t}^{\upsilon }$, $\zeta_{t}^{x}$, $\zeta_{t}^{y}$, $\zeta_{t}^{z}$, and $\zeta_{t}^{\alpha }$, are usually assumed to be a zero-mean Gaussian.

In a particle filter-based inference algorithm, samples were first drawn according to the dynamics of the state vector and the previous state value, and then, the implicit shape matching defined in Equation (16) was performed to assign a weight for each particle. The mean estimation of weighted samples produces the solution in the present frame. The pseudo-code for the ATR-Seg algorithm is given in Table 2. Thanks to the unique structure of JVIM, we can capture the continuous and smooth shape evolution during target tracking and recognition, where segmentation **Φ***_t_* is also archived as a by-product via the shape-aware level set. We expect that the proposed ATR-Seg algorithm has some advantages over other methods that require pre-processing or feature extraction prior to ATR inference [8,26].

## 7. Experimental Results

This experimental section provides a detailed evaluation of the ATR-Seg algorithm in six parts. First, we briefly talk about training data collection for JVIM learning with some qualitative results of shape interpolation. Second, we introduce the infrared ATR database used in this work and how different shape models are to be evaluated collectively and fairly. Third, we present the results of the particle filter-based infrared ATR algorithm, where four shape models (JVIM, CVIM, LL-GPLVM, nearest neighbor (NN)) are compared in the case of explicit shape matching. Fourth, we discuss the results of the proposed ATR-Seg algorithm, which involves JVIM-based implicit shape matching and is compared with the algorithms using explicit shape matching (with JVIM and CVIM). Fifth, we discuss the target segmentation results, which are the by-product of the ATR-Seg algorithm. We will also discuss some limitation of ATR-Seg along with some failed cases.

### 7.1. Training Data Collection

**Figure 8.**
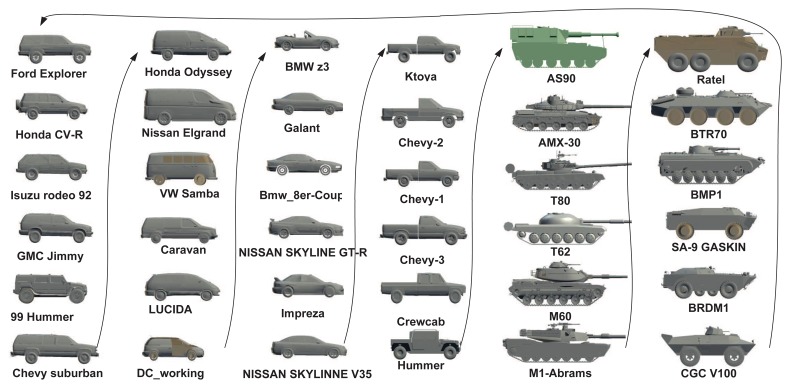
All 36 CAD models used in this work, which are ordered according to the class-constrained shortest-closed-path [8]. (Reprint from [24], with permission from Elsevier.)

In our work, we considered six target classes as [8], *i.e.*, SUVs, mini-vans, cars, pick-ups, tanks and armored personnel carriers (APCs), each of which has six sub-classes, resulting in a total of 36 targets, as shown in Figure 8. These 36 targets were ordered along the view-independent identity manifold according to a unique topology optimized by the class-constrained shortest-closed-path method proposed in [8] (before training). We considered aspect and elevation angles in the ranges 0 ≤ *θ* < 2*π* and 0 ≤ *ϕ* < *π*/4, which are digitized in the interval of *π*/15 and *π*/18 rad, respectively. A total of 150 training viewpoints were used for each target; all training data are generated by their 3D CAD models. In order to reduce the data dimension, the DCT-based shape descriptor proposed in [7] was used here to represent all training shapes for manifold learning. We first detect the contour of a 2D shape (120 × 80) and then apply the signed distance transform to the contour image, followed by the 2D DCT. Only about 10% DCT coefficients are used to represent a shape, which are sufficient for nearly lossless shape reconstruction. Another advantage of this shape descriptor is that we can do zero-padding prior to inverse DCT to accommodate an arbitrary scaling factor without additional zooming or shrinking operations.

**Figure 9.**
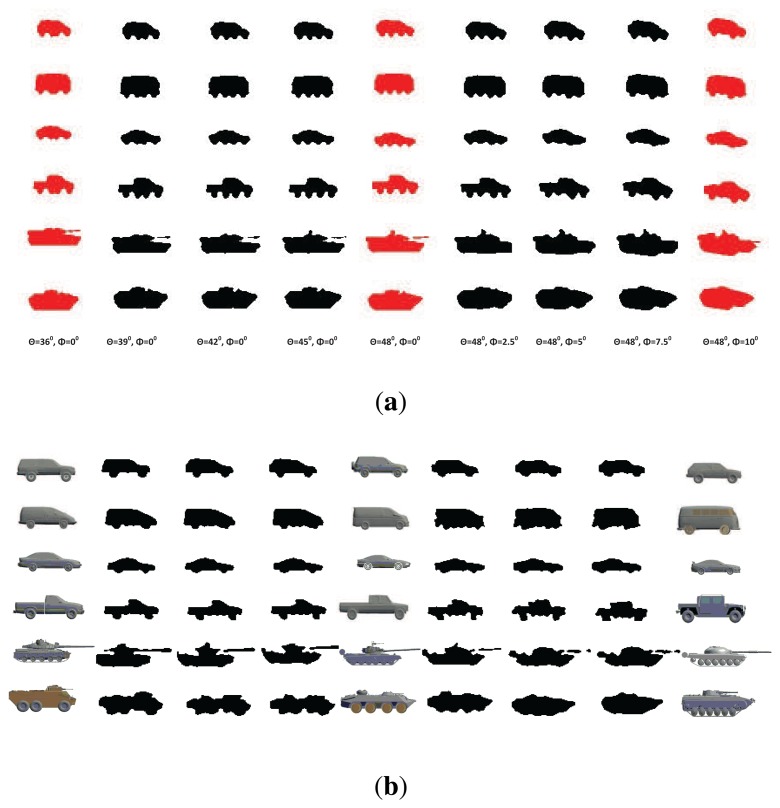
Qualitative analysis of JVIM shape interpolation: (**a**) along six identity-specific view manifolds. (**b**) along the view-independent identity manifold between two training target types. (Reprint from [24], with permission from Elsevier.)

JVIM-based shape interpolation is demonstrated in Figure 9, which manifests its capability of handling a variety of target shapes with respect to viewpoint changes for a known target, as well as the generalization to previously unseen target types. In Figure 9a, we pick one target type from each of the six classes. For each target type, we can obtain an identity-specific view manifold from JVIM along which we can interpolate new target shapes of intermediate views (in black) between two training view-points. A smooth shape transition is observed across all interpolated shapes, despite the strong nonlinearity of training shapes. Figure 9b shows the shape interpolation results (in black) along the view-independent identity manifold for the same side view. Although the interpolated shapes are not as smooth as previous ones, most of them are still meaningful, with a mixed nature of two adjacent training target types along the identity manifold. Compared to CVIM in [8], which assumes that the identity and view manifolds are independent, JVIM shows better shape interpolation results by imposing a conditional dependency between the two manifolds and is also more computationally efficient due to local inference. A detailed comparison can be found in [26], where JVIM is found to be advantageous over CVIM and several GPLVM-based shape models, both qualitatively and quantitatively.

### 7.2. Infrared ATR Database and Shape Models

We have obtained a set of mid-wave IR sequences from the SENSIAC ATR database [11], which includes IR imagery of civilian and military ground vehicles maneuvering around a closed-circular path at ranges from 1–3 km. Forty sequences from eight different target types at ranges of 1.0 km, 1.5 km, 2.0 km, 2.5 km and 3 km were selected for this work. For each sequence, tracking was performed on 500 frames. Background subtraction [30] was applied to each frame for clutter rejection, which is needed for two competing algorithms involving explicit shape matching. For each tracking method, the particle filter was initialized with the ground-truth in the first frame. Similar to [8], the process noise of the heading direction $\zeta_{t}^{\psi }$ is assumed to be a non-zero mean Gaussian to accommodate the circular moving trajectory which is necessary due to the ill-posed nature of image-based 3D tracking. This assumption can be relaxed if 3D pose estimation is not needed. Using the metadata provided with the database and a calibrated camera model, we computed the 3D ground-truth of position and aspect angle (in the sensor-centered coordinate system) for each frame. We refer the readers to [26] for more details about the ATR database.

In the following infrared ATR evaluation, we compare JVIM with LL-GPLVM [9] and CVIM [8], as well as the traditional nearest neighbor shape interpolation (NN). Both JVIM and CVIM treat shape factors (view and identity) continuously. To make a fair comparison, we learned a set of target-specific view manifolds by using LL-GPLVM, which involves a hemisphere as the topology constraint for manifold-based shape modeling. Then, we augment a “virtual” circular-shaped identity manifold (similar to that in JVIM and CVIM) for LL-GPLVM, where a NN method is used to “interpolate” arbitrary target types via training ones. Likewise, two “virtual manifolds” are introduced for the NN-based shape model, where we use the nearest neighbor to find the best matched training shapes. Thus, the two shape variables for four shape models can be inferred in a similar continuous way during ATR inference.

### 7.3. ATR with Explicit Shape Matching

We adopted the particle filter-based ATR algorithm used in [8], where JVIM, CVIM, LL-GPLVM and NN are evaluated in the case of explicit shape matching. In the CVIM-based ATR algorithm, two independent dynamical models are used. In JVIM-based tracking, the dynamic model is a two-stage one, where the first stage is along the view-independent identity manifold, while the second stage along the identity-dependent view manifold. For the LL-GPLVM-based ATR algorithm, one dynamic model is defined on each target-specific view manifold and one on the virtual identity manifold, where NN is used for identity interpolation. For the NN-based ATR algorithm, we employ two dynamic models on two virtual manifolds, like those in CVIM, where shape interpolation is done via NN (*i.e.*, just using the training shapes).

**Figure 10.**
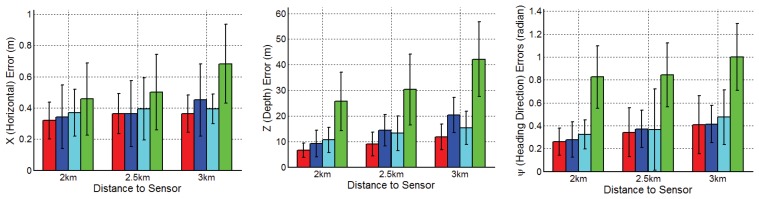
Comparison of the tracking errors of the horizontal position, slant range and heading direction. In each plot, the results for each method averaged over eight target types for each range. From left to right, the plot gives the results for JVIM (first, red), couplet of view and identity manifolds (CVIM) (second, blue), local linear (LL)-Gaussian process latent variable model (GPLVM) (third, cyan) and nearest neighbor (NN) (forth, green). (Reprint from [24], with permission from Elsevier).

The ATR performance of four shape models was evaluated with respect to three figures of merit: (1) **p***_x_* (horizontal) position error (in meters); (2) **p***_z_* (slant range) position error (in meters); and (3) heading direction error *ψ* (in rads). Quantitative tracking performance results are reported in Figure 10, which give the horizontal, slant range and heading direction tracking errors, respectively, averaged over the eight target types for each range. It is shown that JVIM gains 9%, 10% and 35% improvements over CVIM, LL-GPLVM and NN along the horizontal direction, respectively, 35%, 31% and 72% along the slant range, respectively, and 5%, 13% and 62% along the heading direction, respectively. The results demonstrate that JVIM delivers better tracking performance with respect to all three figures of merit, with the advantage over CVIM, LL-GPLVM and NN being particularly significant for the range estimation.

### 7.4. ATR-Seg with Implicit Shape Matching

The proposed ATR-Seg algorithm (noted as Method I in the following) was tested against 15 SENSIAC sequences of five targets (SUV, BMP2, BTR70, T72 and ZSU23) under three ranges (1 km, 1.5 km and 2 km). Two more methods, Method II (JVIM with explicit shape matching, [23,26]) and Method III (CVIM [8]), were considered for comparison. All methods share a similar inference algorithm shown in Figure 7. Both Methods II and III involve explicit shape matching, and JVIM was used for both Methods I and II, while CVIM was used for method III. The tracking results are shown in Table 3. Results for tanks were averaged over T72 and ZSU23, and those for APCs averaged over BTR70 and BMP2. It is shown that Method I outperformed Methods II and III by providing lower tracking errors. More importantly, unlike Methods II and III, which require target pre-segmentation, Method I accomplishes target segmentation along with tracking and recognition as a by-product.

During tracking, the target identity is also estimated frame-by-frame by three methods, and the recognition accuracy is calculated as the percentage of frames where the target types were correctly classified in terms of the six target classes. The overall recognition results of three methods are shown in Table 4, where all methods perform well, and Method I (ATR-Seg) still slightly and moderately outperforms Methods II and III, respectively. Especially, when the range is large, e.g., 2 km, the advantage of Method I over Method III is more significant. This is mainly due to the fact that target segmentation is less reliable when the target is small.

**Figure 11.**
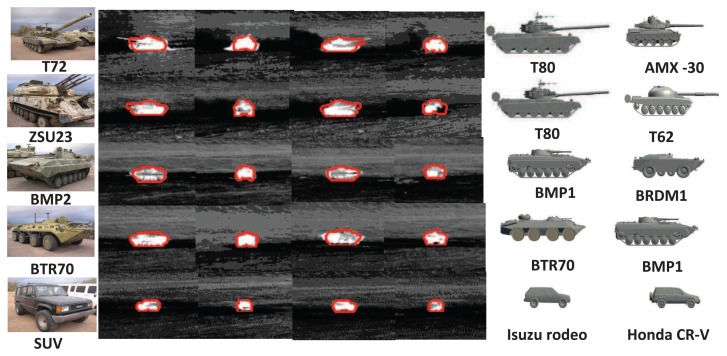
ATR-Seg results for five IR sequences. Column 1: truth target types. Columns 2–5: selected IR frames overlayed with the segmentation results. Columns 6–7: the two best matched training targets along the identity manifold. (Reprint from [28], with permission from IEEE.)

The tracking, recognition and segmentation results of Method I (ATR-Seg) against five 1.5-km sequences were shown in Figure 11, where the two best matched target types are presented to show sub-class target recognition. As shown in Figure 11 (the forth tracking result of ZSU23), part of ZSU23 is missing during tracking; the proposed method still can give an accurate segmentation and tracking result. ATR-Seg uses the intensity information from the present frame to build the energy term in Equation (20) that reduces the error accumulation over time and then evaluates how likely a hypothesized shape created by JVIM can segment a valid target at the predicted position. On the other hand, Method III in [8] uses the background subtraction results and involves an explicit shape comparison for evaluation, so the tracking and recognition results highly depend on the pre-segmentation results.

### 7.5. ATR-Seg Segmentation Results

**Figure 12.**
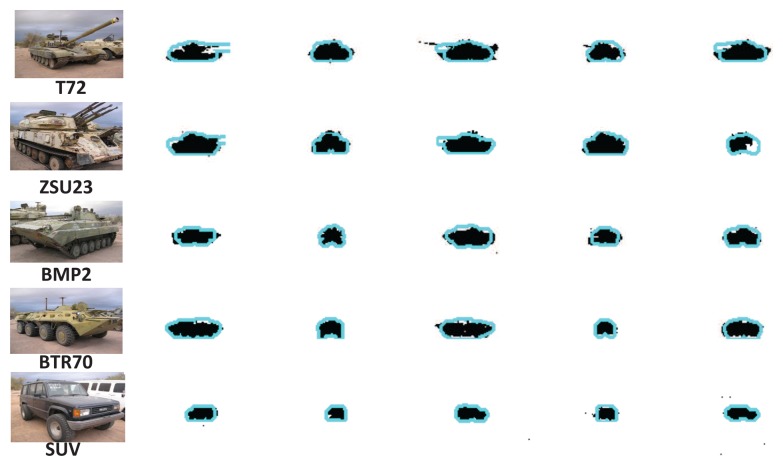
Segmentation results of five targets at the range of 1.5 km.

We evaluated the segmentation performance of ATR-Seg using the metric of the overlap ratio. The ground-truth segmentation results were generated manually for five randomly selected frames in each of 15 sequences. For a comparison, we also computed the overlap ratios for background subtraction results, which are averaged around 81%. While those of ATR-Seg are averaged around 85%. It is worth noting that the segmentation results of ATR-Seg are essentially constrained by the training shapes created from the CAD models, and the training models may have some shape discrepancy with the observed targets in the SENSIAC data. Another source of segmentation errors is due to tracking errors. Some segmentation results of five targets at 1.5 km were shown in Figure 12, where we overlaid the ATR-Seg results (contours) over the ground-truth ones. Background subtraction is not easy for a moving platform and is susceptive to the occlusion problem, while ATR-Seg is more flexible and robust to the sensor ego-motion and has great potential for occlusion handling, due to the shape prior involved [6,7].

### 7.6. Limitation and Discussion

**Figure 13.**
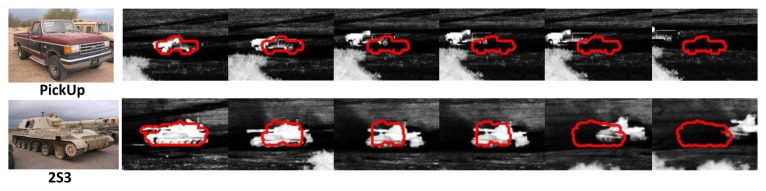
Tracking failure for the pick-up and 2S3 sequences at 1.5 km.

There are two limitations of ATR-Seg due to the unsupervised nature of the level set, where no prior is used for foreground/background pixel intensities, and the mismatching between the training targets and the test ones. Thus, when a major part of a target is occluded or part of a target is similar to the background, the shape-aware set will lose the sensitivity for segmentation evaluation, leading to tracking failure, as shown in Figure 13 (first row), which shows the failed results for the pick-up sequence at 1.5 km. The mismatching and the low-quality data are the main reasons for the tracking failure of 2S3 at a range of 1.5 km (second row in Figure 13). One possible remedy is to incorporate some pixel priors of background and foreground into the level set energy function. However, an online learning scheme may be needed to update the pixel priors that are usually necessary for a long infrared sequence [31]. It is worth emphasizing that the goal of this work is to test the potential of a “model-based” approach that only uses CAD models for training. It is a natural extension to incorporate real infrared data for training that is likely to improve the algorithm robustness and applicability significantly.

## 8. Conclusion

A new algorithm, called ATR-Seg, is proposed for joint target tracking, recognition and segmentation in infrared imagery, which has three major technical components. First is a novel GPLVM-based shape generative model, the joint view-identity manifold (JVIM), which unifies one view-independent identity manifold and infinite identity-dependent view manifolds jointly in a semantically meaningful latent space. Second is the incorporation of a shape-aware level set energy function that evaluates how likely a valid target can be segmented by a shape synthesized by JVIM. Third, a particle filter-based sequential inference algorithm is developed to jointly accomplish target tracking, recognition and segmentation. Specifically, the level set energy function is used as the likelihood function in the particle filter that performs implicit shape matching, and a general motion model is involved to accommodate a highly maneuvering target. Experimental results on the recent SENSIAC ATR database manifest the advantage of ATR-Seg over two existing methods using explicit shape matching. This work is mainly focused on a shape-based approach. One possible future research issue is to involve other visual cues, such as pixel intensities or textures, to enhance the sensitivity and discriminability of the shape-aware level set energy function, which could mitigate the limitations of the ATR-Seg algorithm.

## Figures and Tables

**Table 1.** Descriptions of all mathematical symbols.

Symbols used in Problem Formulation (Section 3)	

**p**	the target's 3D position **p** = [*p_x_*, *p_y_*, *p_z_*]*^T^*
*α*	the target type
*φ*	the view angle
**Θ**	shape related variables ([*α*, *φ*])
**Φ**	a target shape segmentation
**I***_t_*	an observed image frame at time *t*

Symbols used in JVIM-based shape modeling (Section 4)	

**Y**	JVIM training data
**X**	JVIM latent space
*θ*	the aspect angle of a target
*ϕ*	the elevation angle of a target
*β*	the kernel hyper-parameters of JVIM
*d*	the dimension of the shape space
**w**	the LLE coefficients for local topology encoding
*L_JVIM_*	the JVIM objective function
*L_D_*	the data term in *L_JVIM_*
*L_T_*	the topology term in *L_JVIM_*
**K***_Y_*	the covariance matrix of JVIM learning
**x***_r_*	a reference latent point in JVIM learning
**X***_R_*	the neighborhood of **x***_r_* for local learning
*M*_1_	the size of **X***_R_* (the range of local learning)
**Y***_R_*	the corresponding shape for **X***_R_*
*N*	the size of training data
**x′**	a new latent point for JVIM-based shape interpolation
**X′**	the neighborhood of **x′** for local inferencing
*M*_2_	the size of **X′** (the range of local inferencing)
**Y′**	the corresponding shape data for **X′**
*k*(**x**_1_, **x**_2_)	a RBF kernel function in JVIM
***μ̂**_x′_*	an interpolated shape at **x′** via JVIM
${\hat{\sigma }}_{x^{\prime }}^{2}$	uncertainty of shape interpolation at **x′**

Symbols used in shape-aware level set (Section 5)	

*x*	a 2D pixel location in an image frame
*y*	a pixel intensity value
*M*	foreground/background models *M* = {*M_f_*, *M_b_*}
*H_ϵ_* [·]	the smoothed Heaviside step function

Symbols used in sequential inference (Section 6)	

*ψ_t_*	the heading direction of a ground vehicle in frame *t*
*υ_t_*	the target velocity along *ψ_t_* in frame *t*
**Δ***t*	the time interval of two adjacent frames
**Z***_t_*	the state vector in frame *t* ( ${\text{Z}}_{t}={\left[{\text{p}}_{t}^{T},\upsilon_{t},\psi_{t},\alpha_{t}\right]}^{T}$)

**Table 2.** Pseudo-code for ATR-Seg algorithm.

• Initialization: Initialize the target position, **p**_0_, type *α*_0_, heading direction *ψ*_0_ and speed *υ*_0_ according to the ground-truth and get the initial state, **Z**_0_. Draw ${\text{Z}}_{0}^{j}∼N(Z_{0},1),\forall j\in \{1,\cdots ,N_{p}\}$, *N*_*p*_ is the number of particles.
• For *t* = 1,…, *T* (number of frames)
1. For *j* = 1, …, *N_p_*
1.1 Draw samples ${\text{Z}}_{t}^{j}∼p({\text{Z}}_{t}^{j}|{\text{Z}}_{t-1}^{j})$ as in Equation (21).
1.2 Generate the target shape according to the target state using Equations (9) and (10).
1.3 Compute weights $w_{t}^{j}=p({\text{z}}_{t}|\alpha_{t}^{j},{\text{Z}}_{t}^{j})$ using Equation (16).
End
2. Normalize the weights, such that $\sum_{j=1}^{N_{p}}w_{t}^{j}=\text{1.}$
3. Compute the mean estimates of the target state, ${\hat{Z}}_{t}=\sum_{j=1}^{N_{p}}w_{t}^{j}Z_{t}^{j}$
4. Set ${\text{Z}}_{t}^{j}=\text{resample}({\text{Z}}_{k}^{j},w_{k}^{j})$ to increase the effective number of particles [29].
• End

**Table 3.** Tracking errors for three ATR methods (Method I/Method II/Method III). (Reprint from [28], with permission from IEEE).

**Range**	**Error in**	**Tank**	**APC**	**SUV**	**Total**
1 km	**p***_x_* (*m*)	0.22/0.25/0.22	0.22/0.25/0.18	0.16/0.17/0.19	0.21/0.23/**0.20**
	**p***_z_* (*m*)	5.06/8.67/7.53	4.19/4.48/5.14	9.03/8.36/10.95	**5.51**/6.93/7.26
	*ψ* (*rad*)	0.13/0.17/0.18	0.15/0.32/0.15	0.11/0.24/0.22	**0.13**/0.24/0.18
1.5 km	**p***_x_* (*m*)	0.24/0.19/0.18	0.15/0.21/0.20	0.16/0.56/0.60	**0.27**/0.27/0.27
	**p***_z_* (*m*)	4.40/7.20/7.28	4.70/5.88/5.96	– – NA – –	**4.55**/6.54/6.26
	*ψ* (*rad*)	0.16/0.22/0.24	0.18/0.53/0.51	0.11/0.32/0.35	**0.20**/0.36/0.37
2 km	**p***_x_* (*m*)	0.31/0.27/0.28	0.23/0.19/0.36	0.13/0.17/0.35	0.24/**0.22**/0.32
	**p***_z_* (*m*)	8.68/10.6/8.58	8.95/9.28/7.95	5.35/8.09/14.25	**8.19**/9.55/9.46
	*ψ* (*rad*)	0.19/0.38/0.26	0.41/0.18/0.38	0.08/0.41/0.31	**0.26**/0.31/0.32

**Table 4.** Recognition accuracy (%) for Methods I, II and III. (Reprint from [28], with permission from IEEE).

**Targets**	**Tanks**	**APCs**	**SUV**	**Total**
1 km	100/96/96	100/100/94	100/100/100	**100**/98/96
1.5 km	98/96/94	99/100/89	100/100/100	**99**/98/93
2 km	98/92/86	100/100/85	100/100/98	**99**/98/88
